# Fabrication and Characterization of Functional Biobased Membranes from Postconsumer Cotton Fabrics and Palm Waste for the Removal of Dyes

**DOI:** 10.3390/ijms24076030

**Published:** 2023-03-23

**Authors:** Mohd Jahir Khan, Zoheb Karim, Boonya Charnnok, Thiprada Poonsawat, Pattaraporn Posoknistakul, Navadol Laosiripojana, Kevin C.-W. Wu, Chularat Sakdaronnarong

**Affiliations:** 1 Department of Chemical Engineering, Faculty of Engineering, Mahidol University, Nakhon Pathom 73170, Thailand; 2 MoRe Research Örnsköldsvik AB, SE-89122 Örnsköldsvik, Sweden; 3 Department of Specialized Engineering, Energy Technology Program, Faculty of Engineering, Prince of Songkla University, Hat Yai, Songkla 90110, Thailand; 4 Department of Science and Bioinnovation, Department of Science, Faculty of Liberal Art and Science, Kasetsart University, Kamphaeng Saen, Nakhon Pathom 73140, Thailand; thiprada@ku.th; 5 The Joint Graduate School of Energy and Environment, King Mongkut’s University of Technology Thonburi, 126 Pracha Uthit Road, Bang Mot, Thung Khru, Bangkok 10140, Thailand; 6 Department of Chemical Engineering, National Taiwan University, No. 1, Sec. 4 Roosevelt Road, Taipei 10617, Taiwan; 7 Center of Atomic Initiative for New Materials (AI-MAT), National Taiwan University, Taipei 10617, Taiwan; 8 International Graduate Program of Molecular Science and Technology, National Taiwan University (NTU-MST), Taipei 10617, Taiwan; 9 Department of Chemical Engineering and Materials Science, Yuan Ze University, Chung–Li District, Taoyuan 320, Taiwan; 10 Yonsei Frontier Lab, Yonsei University, 50 Yonsei-ro, Seodaemun-gu, Seoul 03722, Republic of Korea

**Keywords:** cellulose nanofibers, palm waste, postconsumer cotton waste, cellulose membranes, dye removal, sustainability

## Abstract

Textile industries currently face vast challenges for the active removal of colored wastewater. Indeed, sustainable, recyclable, and green approaches are still lacking to achieve this aim. Thus, the present study explored the utilization of highly functional, green, recyclable, fully bio-based, and cost-effective composite membranes from post-consumer cotton fabrics and palm waste for wastewater treatment purposes. Highly functional cellulose nanofibers (CNF) were produced from waste cotton fabrics and filter paper using an acid hydrolysis technique. The yield of nanofibers extracted from waste cotton fabrics and filter paper was 76.74 and 54.50%, respectively. The physical, chemical, and structural properties of nanofibers were studied using various advanced analytical techniques. The properties of isolated nanofibers were almost similar and comparable to those of commercial nanofibers. The surface charge densities were −94.0, −80.7, and −90.6 mV for the nanofibers of palm waste, cotton fibers, and filter paper, respectively. After membrane fabrication using vacuum and hot-pressing techniques, the characteristics of the membrane were analyzed. The results showed that the average pore size of the palm-waste membrane was 1.185 nm, while it was 1.875 nm for membrane from waste cotton fibers and filter paper. Congo red and methylene blue dyes were used as model solutions to understand the behavior of available functional groups and the surface ζ-potential of the membrane frameworks’ interaction. The membrane made from palm waste had the highest dye removal efficiency, and it was 23% for Congo red and 44% for methylene blue. This study provides insights into the challenges associated with the use of postconsumer textile and agricultural waste, which can be potentially used in high-performance liquid filtration devices for a more sustainable society.

## 1. Introduction

Industries are the main cause of pollution in ecosystems. According to reports, there will be a two-fold increase in industrial effluent by 2025, which will lead to more contaminated freshwater resources [1]. The textile and leather industries, among others, use a lot of water and produce a huge volume of colored effluents, which comprise a variety of organic and inorganic compounds as well as poisonous elements. Many synthetic colors, heavy metals, and inorganic chemicals included in textile effluents make them extremely toxic and cancer-causing [1]. Due to their improper handling, textile effluents are the principal contributors to water pollution in rivers and on land. The textile industry relies on manufactured fabrics to use dyes and chemicals [2,3,4]. Cotton fabric is commonly dyed with reactive pigments. During the dying process, 10–15 percent of reactive dyes are lost and end up in the effluent. Due to their chemical makeup, dyes have a high degree of stability and resistance to biological and chemical degradation [5]. Furthermore, industrial wastewater changes the physical, chemical, and biological characteristics of the receiving water bodies. Thus, it is required to remove the colors from the industrial effluent. Some traditional methods, such as adsorption, photocatalytic degradation, chemical oxidation, coagulation/flocculation, and biological treatment, are implemented, but all these highlighted methods have some limitations, such as high energy input in photocatalysis, side stream development in biological treatments, recycling/regeneration in adsorption, and low removal efficiency in coagulation/flocculation, etc. Another challenge is imperfect targeting of nano-pollutants (e.g., dyes, heavy metal ions, toxins, and nanosized living organisms such as bacteria and viruses) [6,7].

Therefore, an urgent need is the search for novel, operative, and cost-effective approaches for the removal of nano-sized pollutants such as dyes. In the literature, pressure-derived synthetic membranes have been used for the removal of colored compounds but high energy consumption (because of high applied pressure, for example, >90 bar for RO) and synthetic polymers used for the production of membranes make this approach less sustainable [6,7]. Thus, in this study, the concept of “functional biobased membrane” has been introduced. To make the membranes completely biobased, sustainable, green, and recyclable, nanocellulose (NC) was used as a functional entity to produce membranes. It is a well-known fact that biopolymers are promising building blocks for preparing materials with excellent performance due to their high efficiency, sustainability, and environmental friendliness. Polymers isolated from plant sources such as polysaccharides (cellulose, starch, chitin, and chitosan) have been used for the fabrication of composite membranes [8,9]. Biomaterials derived from biopolymers have received much attention for a variety of industrial and biomedical applications, including biofluid and water purification [10,11]. In this context, cellulose nanofibers (CNF) are one of the foremost choices in filtration because of their excellent mechanical properties, low density, ease of processability into bulk materials, and versatile surface chemistry [12]. The high functionality of nanocellulose makes it suitable for the production of membrane frameworks specialized in the removal of pollutants such as dyes [13,14].

Therefore, the aim of the present study is to (1) introduce the concept of functional membranes, to achieve this highly functional CNF from postconsumer cotton fabrics (CF) and Whatman filter paper (WFP) via acid hydrolysis, and (2) to fabricate highly functional membranes in which CNF as a functional and structural entity is used. Furthermore, membranes were produced using a scalable process, vacuum filtration followed by pressure drying. Various physical and chemical characterizations of fibers and membranes were studied. Moreover, the surface morphologies of membranes were studied with scanning electron microscopy (SEM). The water permeabilities of the membranes were calculated, and the influence of the pore size distribution and porosity was determined. Methylene blue and Congo red were selected as model pollutants because they are present in the effluent discharged from the textile, paper, and leather industries. The effect of the charge density on the adsorption performance of membranes and the mechanism of dye removal was evaluated using the direct flow mode in a dead-end filtration setup. Therefore, we believe that the outcomes of this study could be utilized for the development of more sustainable functional membranes for the separation of pollutants such as heavy metal ions, pharmaceutical toxins, and living microorganisms such as bacteria and viruses in the future.

## 2. Results and Discussion

The aim of this work was to synthesize environmentally friendly, functional, biobased membranes from CNF extracted from bioresource materials. The raw materials used to produce the functional entity (CNF) are low in cost and easily available. Acid hydrolysis was applied for the extraction of CNF. The chosen procedure could be easily controlled by changing the concentration of solvents/chemicals, which definitely changes the degree of polymerization, crystalline properties, particle size distribution, and thermal properties of the produced CNF. Apart from producing highly valuable membranes, this study minimizes the environmental pollution caused by the disposal of cotton and palm waste. Therefore, the concept of high-value product development from waste has been introduced. Denim jeans and oil palm empty fruit bunch (EFB) waste was used for the production of a valuable functional entity, CNF. Furthermore, this functional entity was further used for the fabrication of highly robust membranes that could be used effectively in the elimination of dyes.

### 2.1. Synthesis and Characterization of Cellulose Nanofibers

A comparative analysis of nanofibers from EFB, CF, and WFP is shown in Table 1. The yield of CNF isolated from CF was 76.74%, remarkably higher than WFP (52.50%). The lower CNF yield from WFP paper might be due to the small fiber size, the high amount of nanocrystal formation that remained in the solvent phase, and loss during washing and separation. Cellulose nanocrystals extracted from cotton fabrics yielded 61% when the fabric was 100% cotton, but it dropped to 55% when mixed cotton fabrics were used for sulfuric acid hydrolysis. Additionally, it has been reported that an initial fabric size in the range of ∼5–10 mm has no significant effect on hydrolysis reactions [15]. The yield of cellulose whiskers was 64% when 11 wt% cotton fibers were hydrolyzed with a 65 wt% aqueous sulfuric acid solution at 45 °C under mechanical stirring for 45 min [16]. The present study showed a significantly greater CNF yield of 76.74 and 52.50% from cotton fiber and Whatman filter paper, respectively, when compared with previous work reporting a 46% yield of cellulose nanocrystals produced by the hydrolysis of waste cotton cloth fibers using mixed acids [17]. Furthermore, a 60.41% cellulose nanocrystal yield was reported when cotton cloth was oxidized with sodium chlorite before acid hydrolysis [18]. The detailed characterizations of isolated CNF from raw materials are mentioned later and further summarized in Table 1.

**Figure 1:**
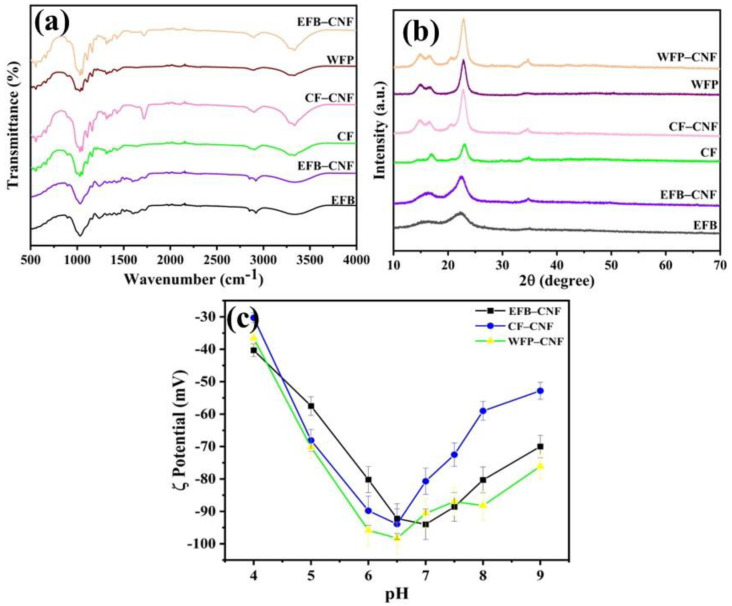
(**a**) FT–IR spectra of palm waste, cotton fabric, filter paper, and extracted nanofibers in the range of 4000 to 500 cm¯^1^ of wavenumber indicating the change in functional group intensity, (**b**) XRD patterns of palm waste, waste cotton fabrics, filter paper, and extracted nanofibers, (**c**) ζ–potential of nanofibers extracted from palm waste (square black dot), waste cotton fabric (blue circle), and Whatman filter paper (yellow triangle) as a function of pH.

FT-IR spectroscopy was used to examine any change in the functional groups present in the initial source materials and final products (Figure 1a). The broad absorption peaks detected at 3325 cm^−1^ in EFB and 3327 cm^−1^ in EFB-CNF were related to the stretching of cellulose hydroxyl groups [19,20]. Peaks around 2880 cm^−1^ were assigned to the aliphatic saturated C-H stretching vibration of cellulose [21]. Absorption in the region of 1800–600 cm^−1^ was called the fingerprint area of the spectra because of various functional groups of EFB. Peaks at 1033 cm^−1^ in EFB-CNF and 1027 cm^−1^ in EFB were attributed to the C-O stretching vibrations of cellulose, while the absorption peak at 1517 cm^−1^ in EFB was because of C=C stretching of hemicellulose and lignin, which did not appear in CNF, as hemicellulose and lignin were removed during the alkaline treatment and bleaching process [22]. The IR spectra of CNF synthesized from CF showed an O-H stretching band at 3747–3112 cm^−1^ with sharpening at 3331 cm^−1^ and multiple peaks in the region 1200–1000 corresponding to the C-O-C stretching vibration of the pyranose ring [23]. All peaks of the CNF spectra were associated with the cellulose structure, and no relevant difference was observed between the spectra of CNF and cotton fiber. The IR spectra of WFP and WFP-CNF displayed O-H stretching at 3332 and 3336 cm^−1^, respectively. A small sharp peak appearing at 1640 cm^−1^ can be attributed to the bending mode of absorbed water in cellulose. The C-O-C pyranose ring was attributed to the structure of cellulose and its stretching vibration was at 1054 cm^−1^ [24]. Thus, using IR spectra it could be easily concluded that no structural change in the cellulose at the molecular level was obtained and changes in the functional groups of native cellulose found in this study were in good agreement with the literature.

The XRD analysis of the raw materials (EFB, CF, and WFP) and isolated CNF is shown in Figure 1b. The crystallinity index (CI) was determined through the difference in crystalline and amorphous constituents in the material. The highest percent of crystallinity was recorded with WFP-CNF (61.18%) followed by CF-CNF (56.17%), while EFB-CNF represents a minimum crystallinity of 56.11%. The lower crystallinity of EFB-CNF could be because of the method used for synthesis. EFB-CNF was synthesized with mechanical grinding, which retains both the amorphous and crystalline portion in the final product [25]. The crystallite size of the nanofibers was calculated by employing the Scherrer equation. The obtained crystallite sizes of the nanofibers were 26.99 nm, 25.38 nm, and 6.52 nm for EFB-CNF, CF-CNF, and WFP-CNF, respectively. Both commercial and synthesized CNF showed characteristic peaks at 2θ of 14.8, 16.5, 22.7 and 34.2° corresponding to the lattice planes ($1\bar{10}$), (110), (200), and (004), respectively, of I_β_ cellulose [26]. The peaks at 16.5 and 22.7° were sharper for CF-CNF and WFP-CNF, while these peaks in EFB were broader. The high crystallinity index obtained for WFP-CNF was attributed to a reduction in its amorphous region. The amorphous region from WFP was removed because of acid hydrolysis, which prompted the hydrolytic cleavage of glycosidic bonds, ultimately releasing individual crystallites [27,28].

The surface ζ-potential of cellulose nanofibers was determined as a function of pH, as shown in Figure 1c. The surface potential was significantly negative for all three nanofibers extracted from different sources. The maximum ζ-potential at neutral medium (pH 7.0) was found as −94.0 ± 4.70 mV for EFB-CNF followed by WFP-CNF (−90.6 ± 4.53 mV). The ζ-potential of CF-CNF was −80.7 ± 4.04 at pH 7.0. The surface ζ-potential was used as an indicator of membrane performance [29]. The impact of the surface ζ-potential was explored with respect to the removal of charged water pollutants (dyes and metal ions). The obtained results confirmed the direct connection between more negative surface ζ-potentials of functional materials for effective membrane separation [29,30].

**Figure 2:**
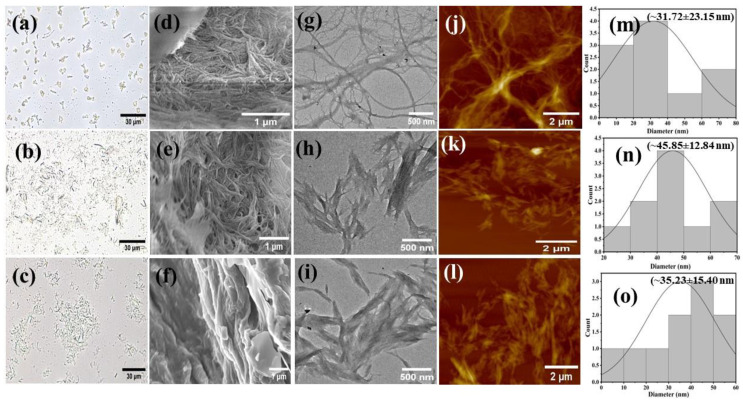
Microscopic characterization of CNF isolated from palm waste, waste cotton fabric, and filter paper. Optical microscopic images of CNF isolated from palm waste (**a**), postconsumer cotton fabrics (**b**), and Whatman filter paper (**c**). Field emission scanning electron microscopic (FESEM) images captured at an acceleration voltage of 5.0 kV of cellulose nanofibers isolated from palm waste (**d**), cotton waste (**e**), and filter paper (**f**). Field emission transmission electron micrographs (FETEM) at an accelerating voltage of 300 kV of empty fruit bunch nanofibers (**g**), cotton waste nanofibers (**h**), and Whatman filter paper nanofibers (**i**). Nanofibers characterized with atomic force microscopy (AFM) of empty fruit bunch nanofibers (**j**), cotton waste nanofibers (**k**), and Whatman filter paper nanofibers (**l**). Nanofiber width distributions measured from FETEM images for empty fruit bunch nanofibers (**m**), cotton waste nanofibers (**n**), and Whatman filter paper nanofibers (**o**).

The shape, size, and surface morphology of commercial and isolated CNF were studied with advanced microscopic techniques (Figure 2). Optical microscopy was carried out to check the progress of nanofiber synthesis, and the result showed that both the cotton fabrics and Whatman filter paper were successfully converted into nanofibrous structures (Figure 2b,c). Furthermore, these images showed highly dispersed fibers in suspension with some aggregation. The surface morphology of CNF was further evaluated with FESEM. It clearly demonstrated that the size and width of commercial nanofibers (Figure 2d) were significantly larger than the nanofibers extracted from CF (Figure 2e) and WFP (Figure 2f). SEM images showed that the CNF had rough surfaces with a disrupted outer layer, which can be attributed to the removal of hemicellulose, waxes, and other impurities during the alkali treatment and bleaching process [17]. CNF produced from different sources exhibited homogeneously well-dispersed morphology. The diameter of the nanofibers was further inferred by processing the FETEM images (Figure 2g–i), which gave clear insight into the resultant CNF morphology. The average diameter of CNF was 31.72 nm, 45.85 nm, and 35.23 nm for EFB-CNF, CF-CNF, and WFP-CNF, respectively (Figure 2m–o).

An AFM study was furthermore carried out for the accurate measurement of nanofiber thickness. The well-distributed CNF measured with AFM are shown in Figure 2j–l. From the FETEM and AFM images, it was confirmed that the nanofiber diameter was maximum for EFB-CNF (~31.72 ± 23.15 nm) compared to CF-CNF (~45.85 ± 12.84 nm) and WFP-CNF (~35.23 ± 15.40 nm). Furthermore, fibers extracted from CF and WFP displayed some crystalline structure, which was in good agreement with the XRD study. By comparing microscopic and nanoscopic images among FESEM, FETEM, and AFM, it could be deduced that nanofibers were successfully produced from both the cotton waste and filter paper.

**Figure 3:**
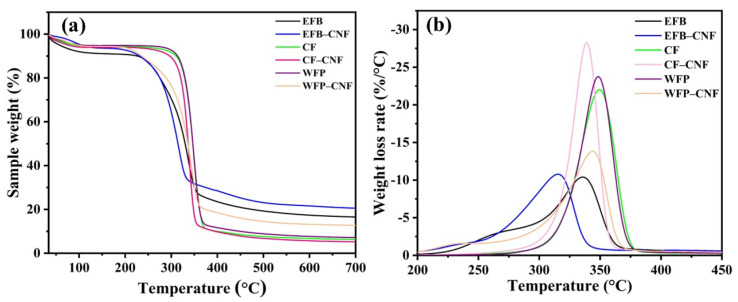
Thermogravimetric analysis (**a**) and derivative thermogravimetric curves (**b**) of source materials (palm waste, postconsumer cotton fabric, and filter paper) and isolated cellulose nanofibers.

Thermal stability of nanofibers is an important characteristic where the operating temperature is high, especially in the synthesis of bio-composites and further application in wastewater treatments [31]. Thermogravimetric analysis (TGA) and derivative thermogram (DTG) curves for extracted nanofibers and resource materials were determined and are shown in Figure 3a, and b, respectively. Table 2 demonstrates the initial and final decomposition temperatures of the nanofibers and source materials. As shown in image 3a, degradation with respect to increased temperature of all reported raw materials and isolated CNF indicates a common slope of curves, as reported in previous published article [32]. The cellulose decomposition of EFB, CF, and WFP began at 293.9, 327.6 and 328.4 °C, respectively (Figure 3a and Table 2). This exhibited that either the degree of molecular arrangements or crystallinity of CF and WFP was higher than EFB, and therefore, it required high heat energy for degradation [33]. The maximum degradation temperatures for EFB, CF, and WFP were 335.8, 349.5 and 348.6 °C, respectively (Table 2). The thermal properties of the synthesized CNF showed that the degradation started at 321.9 °C for CF-CNF, followed by WFP-CNF, which was 308.2 °C, and was lowest for EFB-CNF (277.9 °C). Nevertheless, the maximum degradation temperature was found in a respective degree for WFP-CNF (343.9 °C), CF-CNF (339 °C), and EFB-CNF (315.7 °C), in which EFB-CNF exhibited the lowest maximum degradation temperature among all CNF samples (Figure 3b and Table 2). Degradation of cellulosic components occurs as a result of decarboxylation, depolymerization, and decomposition of cellulose and hemicellulose fragments. Random cleavage of glycosidic linkages in cellulose occurs generally at 275 and 350 °C, which results in reducing the degree of polymerization and therefore, the formation of CO_2_, H_2_O, and a variety of hydrocarbon derivatives. The crystalline order of cellulose fibers affects thermal stability, which decreases after the substitution of cellulose hydroxyls with organic acids [34,35]. According to the result in the present study (Figure 3a, Table 2), among these samples the char residue was lowest in CF and CF-CNF followed by WFP and WFP-CNF and EFB-CNF and EFB consecutively, which indicates a high purity of the cellulose [36] in cotton fibers, which was presumably greater than WFP and EFB.

### 2.2. Characterization of CNF Membrane Framework

**Figure 4:**
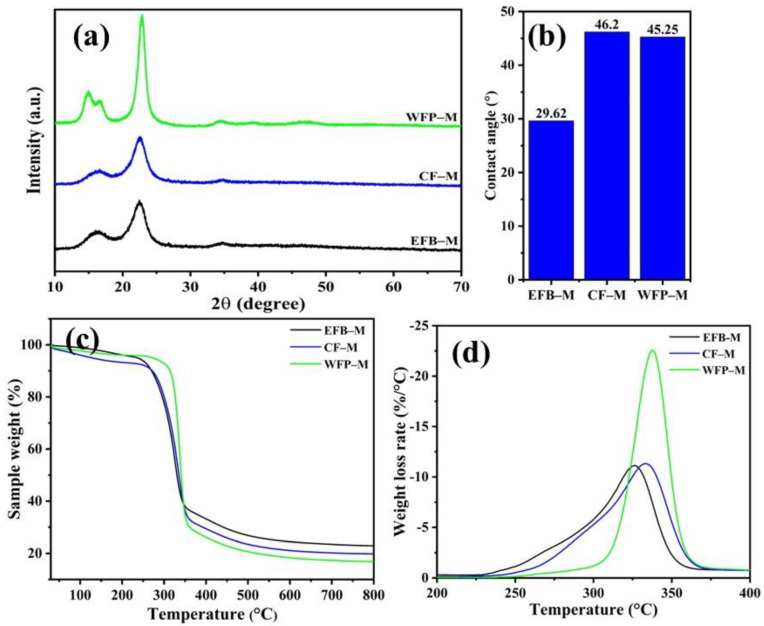
(**a**) X-ray diffractogram of cellulose membranes fabricated from palm waste (EFB–M), postconsumer cotton fabric (CF–M), and filter paper (WFP–M), (**b**) surface hydrophilic properties of fabricated membrane framework determined by water drop test contact angle, and thermal stabilities of membranes evaluated by thermogravimetric analysis (**c**) and derivative thermogravimetric curves (**d**).

The prepared functional CNF membranes were characterized with an XRD analysis to identify any crystallinity changes during fabrication (Figure 4a). The spectra showed peaks that were almost similar to their corresponding nanofibers (Figure 1b). All the membranes showed characteristic peaks at 2θ of 14.8, 16.5, 22.7 and 34.2° attributed to the lattice planes ($1\bar{10}$), (110), (200), and (004), respectively, of I_β_ cellulose [37]. The percent crystallinities of the membranes were 59.52, 65.91 and 73.07% for EFB-M, CF-M, and WFP-M, respectively. The high crystallinity of WFP-M could be responsible for the substantially low crystallite size of isolated CNF compared with other CNFs, as reported in Table 1. Furthermore, it has been mentioned that the WFP has processed fibers that make the isolated WFP-CNF at higher purity relative to the other two raw materials, i.e., CF-CNF and EFB-CNF.

To check the membranes’ performance for the removal of dyes, it is necessary to understand the surface properties of membranes and their interaction with water molecules. The surface hydrophilic/hydrophobic properties of the produced functional CNF membranes were assessed through a water drop test and contact angle measurement. Membranes fabricated from different materials showed the change in water wettability. Water drop contact angles of 29.6, 46 and 45.3° were determined for EFB-M, CF-M, and WFP-M, respectively (Figure 4b). The higher contact angle of CF-M and WFP-M than that of EFB-M displayed more hydrophobic properties of CF-M and WFP-M, which could reduce the removal efficiency of dyes dissolved in water as well as the water flux of membranes. In summary, a high contact angle increases the hydrophobic nature of the membrane and decreases the polarity [38].

The thermal properties of the membranes were determined using the TGA/DTG curve, measured in the temperature range of 30–800 °C (Figure 4c,d). Generally, weight loss above 100 °C is associated with the evaporation of adsorbed water and volatile compounds. Weight loss starting at 290.6, 297.4 and 321.1 °C was found in EFB-M, CF-M, and WFP-M, respectively, which corresponded to the beginning of cellulose decomposition [39] (Figure 4c and Table 3). The DTG analysis demonstrated that the maximum degradation occurred at 326.3 °C for EFB-M, 333 °C for CF-M, and 337.6 °C for WFP-M (Figure 4d and Table 3), which agrees with respective degree of maximum degradation temperature observed in CNFs (Table 2).

**Figure 5:**
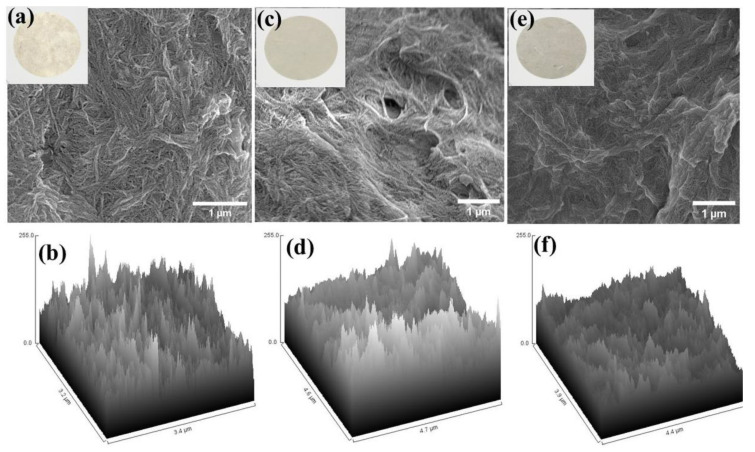
FESEM images of CNF membranes fabricated from palm waste nanofibers (EFB–M) (**a**), cotton nanofibers (CF–M) (**c**), and Whatman filter paper nanofibers (WFP–M) (**e**). Surface roughness of the membranes from FESEM images determined with ImageJ software for EFB–M (**b**), CF–M (**d**) and WFP–M (**f**). Inset showing bright field optical images of EFB–M (**a**), CF–M (**c**), and WFP–M (**e**).

Bright field optical analysis and FESEM analysis of functional CNF membranes were performed to understand their surface morphology and porosity (Figure 5). Bright field optical images confirmed the consistently smooth surface without any drastic changes in morphology and thickness (Figure 5a,c,e, inset). On the other hand, remarkable changes were observed in the surface morphology and roughness when structures were analyzed under an electron microscope (FESEM), which showed an uneven surface thickness and porosity. The surface roughness was further examined with ImageJ software using FESEM images and showed that the maximum surface roughness was observed in EFB-M followed by CF-M, while the minimum was found in the WFP-M (Figure 5b,d,f).

**Figure 6:**
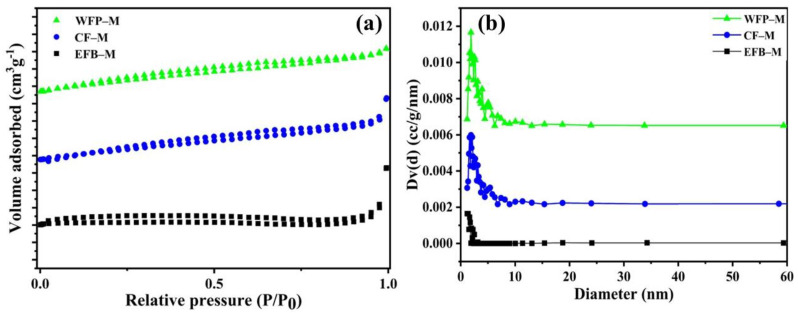
(**a**) Plot of N_2_ adsorption–desorption isotherms of volume adsorbed versus relative pressure (P/P_0_) and (**b**) BJH pore size distributions for the fabricated membranes from palm waste nanofibers, cotton nanofibers, and Whatman filter paper nanofibers.

The specific surface area and pore structure of the membranes were analyzed with BET and BJH studies of N_2_ gas adsorption isotherm (Figure 6). Table 4 summarizes the specific surface area, pore volume, and average desorption pore width of the membranes. The specific surface area was highest for WFP-M (8.19 m^2^ g^−1^) compared to CF-M (5.65 m^2^ g^−1^) and a minimum surface area was observed for EFB-M (0.64 m^2^ g^−1^) (Table 4). The surface area found in the present study was lower than some earlier reports on nanocellulose membrane structures [40,41]. It has been reported that the specific surface area of membrane structure was changed at different drying conditions and chemical treatments. Freeze-dried nano-fibrillated cellulose showed a BET surface area of 26.8 m^2^ g^−1^ whereas the amine-functionalized nano-fibrillated cellulose showed a value of only 7.1 m^2^ g^−1^ [42]. In the current study, vacuum filtration followed by hot pressing was used to produce functional membranes. Indeed, introduced hydrogen bonds during filtration and drying within the network of functional membranes are responsible for the low surface area. The obtained data are further supported by the pore size distribution and porosity. The pore size distributions of the membranes were determined from the BJH method using the Kelvin equation and it was very narrow in EFB-M, with a pore size of 11.85 Å (1.185 nm), which confirmed the dense membrane network. The pore size distribution was broad for CF-M and WFP-M, with an average pore size of 18.73 Å (1.873 nm) for both the membranes. There were no remarkable changes in the pore sizes of the membranes but the pore volume was significantly decreased for EFB-M (0.7 × 10^−2^ cm^3^ g^−1^) compared to both CF-M and WFP-M membranes, having same pore volume (1.3 × 10^−2^ cm^3^ g^−1^).

The water flux of the functional membrane was determined with a dead-end stirred cell at ~0.1 MPa applied pressure maintained using supplied N_2_. Dead-end filtration is also known as frontal filtration. The fluid flow direction is vertical to the membrane surface and the particles are trapped and accumulated on the filter surfaces, while in cross-flow filtration, the fluid to be treated is circulated along a flat, spiral, tubular, or hollow fiber module and the permeate containing water and solutes is collected by filtration across the membrane.

Each membrane was kept in water for 1 h before water flux measurement so that it was fully swollen and wet. The maximum flux was noted for WFP-M as 142.12 L h^−1^ m^−2^, followed by CF-M, which showed 130.75 L h^−1^ m^−2^ (Table 4). The lowest water flux (108 L h^−1^ m^−2^) was recorded for the EFB-M (Table 4). These data were supported by a BET study in which the specific surface area and pore diameter of the membranes were significantly higher for CF-M and WFP-M compared to the EFB-M. The lower water flux of the EFB-M compared to CF-M and WFP-M was apparently because of the narrow pore size distribution, even though EFB-M demonstrated higher hydrophilicity via the water drop test contact angle (Figure 4b). The lower water flux of EFB-M relative to CF-M and WFP-M was mainly because the nanofibers used in EFB-M fabrication had a longer length and smaller width, and thus exhibited stronger networking potential, as shown in the SEM and AFM images (Figure 2), which led to a lower pore volume and pore width of the EFB-M (Table 4). Different pore size distributions in the membranes could be due to high interlocking between the fibers. It is crucial to make a point that BJH pore width and water flux are well-correlated for all produced functional CNF membranes, as reported in Table 4.

### 2.3. Dye Removal Using Fabricated Nanocellulose Membrane

**Figure 7:**
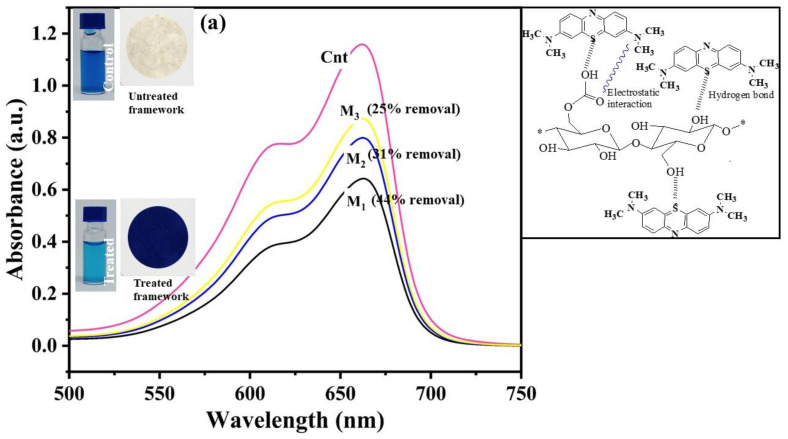

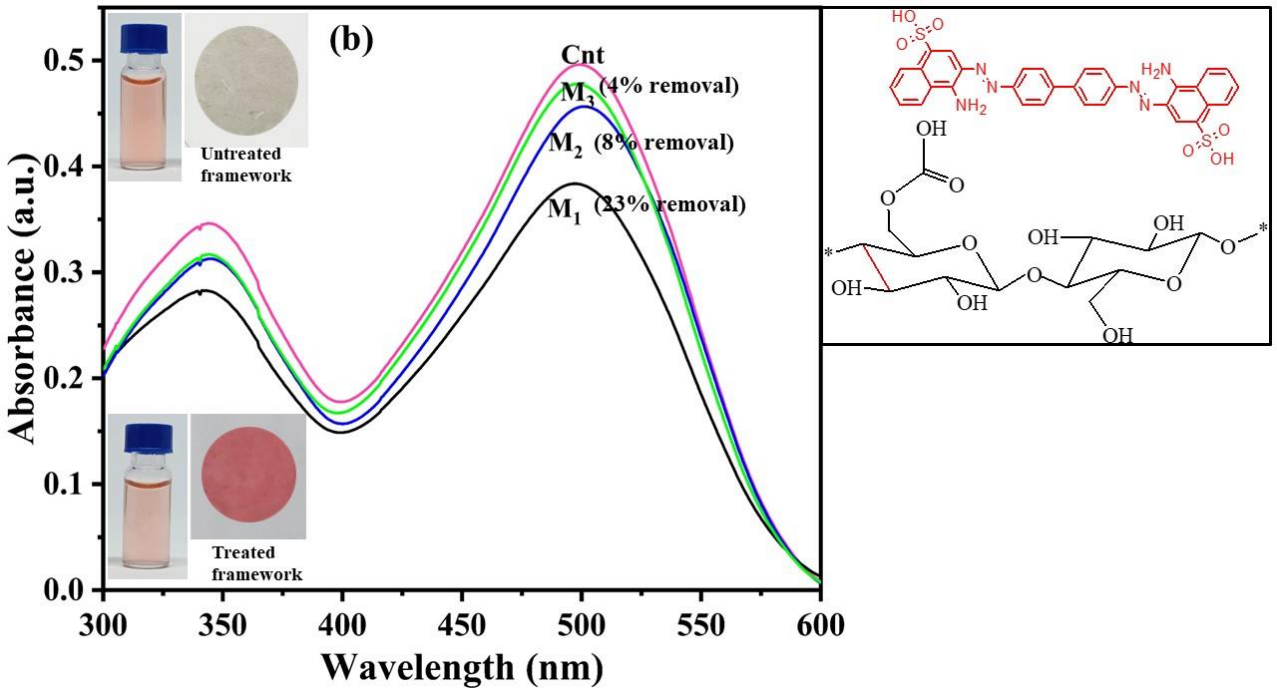
Removal of dyes using functional CNF membranes by direct filtration mode at initial dye concentrations of 10 mg L^−1^. UV-visible spectroscopy showing a decrease in absorbance of dye after dye removal test versus the control (Cnt) at wavelength λ_max_ = 668 nm for methylene blue (**a**) and at wavelength λ_max_ = 478 nm for Congo red (**b**) for membranes fabricated from palm waste (EFB–M or M_1_), cotton fabric (CF–M or M_2_), and Whatman filter paper (WFP–M or M_3_); molecular structure of dyes and their possible interactions with the membranes are shown (right).

From the results, the maximum dye removal efficiency was observed for the EFB-M (M_1_), with 44% removal for methylene blue and 23% removal for Congo red, followed by CF-M (M_2_), which had 31% removal for methylene blue and 8% removal for Congo red (Figure 7). The functional membrane prepared from filter paper (WFP-M or M_3_) was the least effective, as it removed only 25% of the methylene blue and 4% of the Congo red from the dye solutions. The mechanism for dye removal using nanocellulose membranes is apparently adsorption; therefore, hydrogen bonds and ionic interactions between dyes and membranes are the principal factors. Ionic interactions and hydrogen bonds were the predominant forces responsible for the interaction of dyes and cellulose fibers, as depicted in Figure 7. Cellulose nanofibers displayed remarkable negative charges (Table 1) and exhibited a strong interaction with positively charged methylene blue by electrostatic interactions and hydrogen bonding. The molecular size of the dyes could be another reason for the higher dye removal efficacy, since the small-size dye facilitates more adsorbate movement through the membrane pores, thus leaving supplementary adsorption sites available for binding [43]. Methylene blue, with smaller molecular size (13.82 Å or 1.382 nm) [44] compared to Congo red (18.3–25.9 Å or 1.83–2.59 nm) [45], therefore exhibited greater removal percentages in all CNF membranes tested. Moreover, Congo red possesses a negative charge and thus, repulsion occurred between the nanofibers and dye and led to a lower Congo red removal efficiency compared with methylene blue for all membranes investigated.

## 3. Materials and Methods

### 3.1. Materials

Oil palm empty fruit bunch (EFB) cellulose nanofibers (CNF) (99.9% purity) were obtained from Zoep Nano Sdn. Bhd., Malaysia. Formic acid (98/100%) was purchased from Fischer Scientific, Belgium. Sodium chlorite and sodium hydroxide were purchased from Bay Rd, Australia. Glacial acetic acid (100%) was purchased from Merck, Germany. Used cotton pants after cutting into pieces and grinding were taken as a source of post-consumer cotton fabrics. Other chemicals and reagents used in this study were analytical grade and used without any further purifications.

### 3.2. Delignification and Bleaching of Palm Empty Fruit Bunch

The EFB was washed multiple time with distilled water and dried at room temperature. The dried EFB was ground and sieved into +50/−200 mesh. To delignify, EFB (4% *w*/*v*) was treated in a 4% *w*/*v* NaOH solution at 90 °C for 3 h with a constant stirring speed of 200 rpm. After treatment, EFB was separated through vacuum filtration and washed multiple times until the pH of the washing solution reached 7.0. The washed EFB sample was dried in an oven at 40 °C until dry. The delignified EFB sample was bleached by soaking of (4% *w*/*v*) delignified EFB in acetic acid buffer (27 g NaOH and 75 mL glacial acetic acid, diluted to 1 L of distilled water) and 2% *w*/*v* sodium chlorite bleaching solution [46]. The treatment was conducted at 90 °C for 3 h. After bleaching, sample was separated by vacuum filtration and washed several times with deionized water until the pH reached 7.0. The bleached sample was dried in a hot-air oven 40 °C until dry before hydrolysis.

### 3.3. Synthesis of Cellulose Nanofibers

Postconsumer cotton fabric was cut into small square pieces of length <1 cm, separated into yarn, and ground to small fibers for subsequent alkali treatment and bleaching. This was accomplished by soaking 15 g cotton fabric in 300 mL acetic acid buffer (8.1 g NaOH and 22.5 mL glacial acetic acid) and 2% aqueous sodium chlorite. The solution’s pH was approximately 3.6–4.0 [46]. Bleaching was carried out for 2 h at 80 °C, during which cotton fabrics were decolorized and thus made whiter. After bleaching, cellulose fibers were separated from the medium with vacuum filtration and washed several times with deionized water until the pH reached 7.0. The bleached sample was dried at 40 °C in a hot-air oven before hydrolysis.

**Figure 8:**
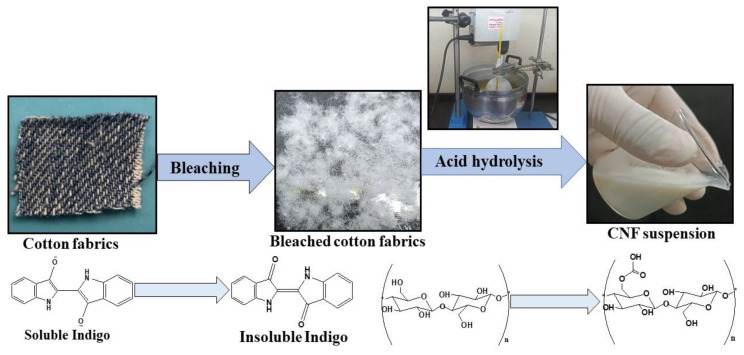
Synthesis of cellulose nanofibers from waste cotton fabrics. The bleaching mixture as discussed in the text was used for the production of long cellulosic fibers and then formic acid was used for the production of CNF.

CNF was synthesized from bleached cotton fabric according to the procedure described in a previous study with some modifications [47]. A schematic representation of cellulose nanofiber synthesis from waste cotton fabrics is shown in Figure 8. A 500 mL round-bottom flask was filled with 300 mL of formic acid, and subsequently bleached cotton fabric (15 g) was added and the mixture was heated at 90 °C and stirred with an overhead stirrer at 1300 rpm for 24 h. After that, the mixture was naturally cooled to room temperature and ultrasonicated for 1 h. To stop the reaction, 800 mL cold, deionized water was added, and the pH was adjusted to 7.0 with 1.0 mol L^−1^ NaOH solution. The obtained suspension was centrifuged at 6000 rpm for 10 min, and the cellulose pellet was further washed with deionized water until the acid was removed completely. The obtained cellulose nanofiber suspension was homogenized with a high-speed homogenizer at 15,000 rpm for 3 h. The final CNF suspension was stored at 4 °C for further use. The same process was followed for the synthesis of cellulose nanofibers from WFP.

### 3.4. Characterization of Cellulose Nanofibers

Fourier-transform infrared spectroscopy (FT-IR) was carried out to evaluate changes in the functional groups in CNF. FT-IR (Nicolet 6700, Thermo Scientific, Waltham, MA, USA) spectra were recorded in the range of 500–4000 cm^−1^, with 64 scans at a resolution of 4 cm^−1^. The measurement was carried out in the attenuated total reflectance (ATR) measurement mode.

The crystalline properties were determined with an X-ray diffraction spectrometer (Bruker D8-Discover, Germany). The analysis was performed with an XRD spectrometer equipped with a Cu tube at 2θ of 20–80° in steps of 0.02° at a scan rate of 1° per step. The crystallinity index of the materials was calculated using the following Equation (1).

(1) $$\mathrm{CI}\left(\%\right)=\frac{I\left(22{}^{\circ}\right)-I\left(18{}^{\circ}\right)}{I\left(22{}^{\circ}\right)}\times 100$$

where CI (%) stands for the crystalline index of cellulose. I(22°) (arbitrary units) represents the peak diffraction intensity corresponding to crystalline cellulose, and I(18°) is the peak diffraction intensity corresponding to the amorphous section in cellulose at 22 and 18°, respectively. The particle size (D) of the samples was determined by Scherer’s formula as follows:

(2) $$D=\left(\frac{0.9\lambda }{B\mathrm{Cos}\theta }\right)$$

where λ is the X-ray wavelength (1.54060 Å), B is the full width at half-maximum intensity of cellulose, and θ is the diffraction angle.

Charges on cellulose nanofibers were evaluated by measuring the ζ-potential in triplicate using Zeta-sizer (Malvern Nano ZS, Malvern, Herrenberg, Germany) at 25°C. The ζ-potential was measured as a function of pH, which was adjusted using 0.05 mol L^−1^ KOH and HCl when the applied electrode voltage spacing of a 16 mm cell was 3.4 V. CNF (0.01%) were ultrasonicated and stirred for 2 min prior to measurements to improve the fiber dispersion. The standard deviation and mean values were calculated from three individual measurements.

The progress of CNF synthesis during acid hydrolysis was analyzed with an optical microscope (Leica, Allendale, NJ, USA). A drop of nanofibers was dried on a microscope slide and imaged with the microscope at different magnifications. The surface morphology of the cellulose nanofibers and fabricated membranes was assessed with a field emission scanning electron microscope (FE-SEM) (FEI Quanta 450, Hillsboro, USA) at an acceleration voltage of 5 kV. CNF and membrane samples were placed on a double-sided tape, sputter-coated with platinum for 10 min, and examined under the microscope.

Shape and diameter of CNF were examined using a field emission transmission electron microscope (FE-TEM, JEOL JEM-3100 F, Tokyo, Japan) at an accelerating voltage of 300 kV. Samples were prepared by adding 10 μL of a 0.01 wt % homogeneous suspension of nanofibers on a copper grid, followed by drying in the air at room temperature.

The surface morphology of CNF was further characterized with tapping mode using an atomic force microscope (AFM; Park systems Model NX10, Rosemont, IL, USA). Fiber samples were placed dropwise on a mica wafer, air dried, and imaged with AFM. The size of nanofibers was determined by processing the TEM images using ImageJ software.

The thermal properties of CNF and their corresponding raw materials were evaluated with a thermogravimetric analyzer (NETZSCH, STA 449 F3 Jupiter, Selb, Germany). Samples (15 mg) were heated from 30 °C to 800 °C at a heating rate of 10 °C min^−1^ under nitrogen gas purge until no weight change was detected.

### 3.5. Fabrication of Functional Cellulose Membranes

The functional membranes were fabricated using vacuum filtration. The produced CNF (10 wt %) slurry was diluted to 1 % *w*/*v* before vacuum filtration. The 100 mL of 1 % *w*/*v* CNF was filtered at 36 kPa under vacuum conditions. The filtered samples after water removal were transferred and then pressed between Teflon plates at 5 kg weight for 47.76 cm^2^ area. Blotting paper was placed above the fabricated membranes for fast drying. This whole setup was placed in an oven at 40 °C for 48 h. The blotting paper was replaced every 10 h. The thickness of the functional membranes, which varied at different volumes and concentrations of CNF, was measured.

### 3.6. Characterization of Functional Cellulosic Membranes

The pore size distribution and surface area of the prepared functional membranes were determined with the N_2_ adsorption method using the Brunauer–Emmett–Teller (BET) (Micromeritics ASAP 2000, Stockholm, Sweden) instrument. The samples were degassed at 80 °C for 24 h in dry N_2_ flow. The surface wettability of membranes was determined with optical contact angle measurements (OCA 15 Plus instrument, Filderstadt, Germany) using the sessile drop technique [48]. Functional membranes were cut into small pieces and fixed to the sample holder. Liquid water was dispensed automatically on the membranes and images of the droplet were captured using high resolution camera at the instant when the drops touched the surface of the membrane.

The water flux of the functional membranes was determined using a dead-end stirred cell (Sterlitech HP4750, Auburn, WA, USA). The produced membranes were cut into circles having diameter of 52 mm and surface area of 21.23 cm^2^ and placed into the dead-end stirred cell. The volume of water passed through the membrane for a certain time interval was determined and the flux was calculated (L h^−1^ m^−2^) through the active filtration area of 21.23 cm^2^.

### 3.7. Removal of Dyes from Model Solution

Methylene blue (cationic) and Congo red (anionic) dyes were chosen to examine the efficacy of the produced functional membranes. It is well known that these dyes are excessively used in the textile and paper industries. The stock concentrations (10 mg L^−1^) of both dye solutions were prepared in distilled water at an acidic pH (pH 5.0). The volume of the dyes used in the dead-end stirred cell was 300 mL for all separation experiments. In order to check the membrane efficiency, the selected concentration (10 mg L^−1^) of both dyes was higher than the range present in real discharged effluent from pulp and paper industries [49,50]. The functional membranes prepared from EFB, CF, and WFP were called M_1_, M_2,_ and M_3_, respectively.

The removal efficiency of the functional membranes was measured in a direct filtration mode and monitored the dyes’ absorbent change using a UV-visible spectrophotometer. The spectra of control and treated dyes were recorded using a spectrophotometer (Shimadzu, UV-1800, Kyoto, Japan). Three independent measurements were carried out for the final calculations. The dye-removal efficiency was calculated according to the following Equation (3):

(3) $$\mathrm{Removal}\left(\%\right)=\frac{\mathrm{Co}-\mathrm{Ct}}{\mathrm{Co}}\times 100$$

where C_o_ and C_t_ are the concentrations (mg L^−1^) of the control and treated dye solutions passing through the membrane, respectively.

## 4. Conclusions

In the current study, we have introduced the concept of valuable product development from biowaste; thus, oil palm empty fruit bunch, filter paper and denim cotton waste were used to produce highly functional cellulosic nanofiber membranes. All the obtained properties of CNF were good enough to produce functional membranes for water purification applications. Therefore, the concept of functional membranes has been proven and demonstrated. The produced functional membranes were further applied for the separation of dyes from model wastewater. Indeed, the functionality of the produced functional membranes has direct impact on the membrane performance, i.e., high surface charge and more negative ζ-potential, which are responsible for the effective separation of dyes from the model textile effluent. Furthermore, the final characteristics of fabricated membranes could be easily tuned by changing the type and concentration of CNF. The main driving force responsible for the separation of dyes is adsorption and ionic interactions (mainly negative–positive interaction between the dye and functional CNF). Thus, all obtained results indicate the feasibility of upscaling of these membranes in the future. We, moreover, believe that some other charged water pollutants such as metal ions, pharmaceutical toxins, and living microorganisms (bacteria and viruses) could also be removed with high efficiency using the developed membranes.

## Figures and Tables

**Table 1:** Physicochemical properties of cellulose nanofibers extracted from palm waste, postconsumer cotton fabric, and Whatman filter paper.

Samples	Yield (%)	Surface ζ-Potential (mV) at pH 7.0	Crystallite Size (nm)	Crystallinity (%)
EFB–CNF	NA	−94.0 ± 4.70	26.99	56.11%
CF–CNF	76.74%	−80.7 ± 4.04	25.38	56.17%
WFP–CNF	52.50%	−90.6 ± 4.53	6.52	61.18%

NA = not applicable.

**Table 2:** TGA and DTG analysis of palm waste, cotton fabric, filter paper, and their final extracted nanofibers.

Samples	TGA and DTG Analysis		
	Onset Degradation Temperature (°C)	Max Degradation Temperature (°C)	Char Residue at 898.4 °C (%)
EFB	293.9	335.8	15.9
EFB–CNF	277.9	315.7	20.1
CF	327.6	349.5	6.1
CF–CNF	321.9	339.0	5.0
WFP	328.4	348.6	7.0
WFP–CNF	308.2	343.9	12.5

**Table 3:** TGA and DTG analysis of fabricated CNF membrane framework.

Samples	TGA and DTG Analysis		
	Onset Degradation Temperature (°C)	Max Degradation Temperature (°C)	Char Residue at 898.4 °C (%)
EFB–M	290.6	326.3	22.74
CF–M	297.4	333.0	19.81
WFP–M	321.1	337.6	16.76

**Table 4:** Surface area, pore volume, pore size diameter, and water flux of CNF membrane framework tested at ~0.1 MPa applied pressure.

Samples	Surface Area (m^2^ g^−1^)	Pore Volume (cm^3^ g^−1^)	BJH Desorption Pore Width (Å)	Water Flux (L h^−1^ m^−2^)
EFB–M	0.64	0.7 ×10^−2^	11.85	108.00
CF–M	5.65	1.3×10^−2^	18.73	130.75
WFP–M	8.19	1.3×10^−2^	18.73	142.12

## Data Availability

All data generated or analyzed during this study are included in this article.
